# Etiology, diagnosis, treatment, and prevention of human papilloma virus-associated oropharyngeal squamous cell carcinoma

**DOI:** 10.1007/s10147-023-02336-8

**Published:** 2023-04-24

**Authors:** Hirotaka Shinomiya, Ken-ichi Nibu

**Affiliations:** Department of Otolaryngology-Head and Neck Surgery, Kobe University Graduate School of Medicine, 7-5-1 Kusunoki-Cho, Chuo-Ku, Kobe, Hyogo 650-0017 Japan

**Keywords:** Human papilloma virus, Oropharyngeal cancer, Vaccination, Clinical trial

## Abstract

Classical oropharyngeal squamous cell carcinoma (OPSCC) caused by alcohol consumption and smoking and HPV-associated OPSCC caused by human papillomavirus (HPV) infection have different etiologies, incidences, and prognoses. Therefore, the 8th American Joint committee on Cancer (AJCC) and Union for International Cancer Control (UICC) TNM classifications propose distinguishing HPV-associated OPSCC from classical OPSCC and classifying it as an independent disease. Therefore, this review provides an overview of HPV-associated OPSCC from the perspectives of epidemiology, carcinogenesis, development, diagnosis, treatment, and prevention. The incidence of HPV-associated OPSCC is increasing. Although HPV vaccination has been shown to be effective at reducing the incidence of cervical cancer, it is still unclear how it affects the incidence of HPV-associated OPSCC. Additionally, the prognosis of patients with HPV-associated OPSCC is extremely favorable compared to that of patients with classical OPSCC. Therefore, patients with HPV-associated OPSCC may undergo reduced-dose therapy, although attempts to reduce treatment intensity should be carefully planned to ensure they do not compromise oncological outcomes, and large-scale trials aimed at reducing treatment intensity are ongoing.

## Introduction

Human papillomavirus (HPV) contributes to the carcinogenesis of various cancers, including cervical, oropharyngeal, and anal cancers. Cervical cancer has long been known to be an HPV-associated cancer. Therefore, HPV vaccination has been introduced to prevent cervical cancer in developed countries. However, the association of HPV with oropharyngeal cancer was recognized only recently, and the full picture of HPV-associated oropharyngeal cancer has been gradually unraveled. Classically, oropharyngeal squamous cell carcinoma (OPSCC) was believed to be caused by alcohol or tobacco use. However, Mork et al. [1] reported an association between HPV infection and OPSCC in 2001, and HPV infection has since been recognized as a significant carcinogenic factor for OPSCC.

Over the past two decades, smoking and alcohol consumption rates have continuously decreased in developed countries. Consequently, the incidence of classical OPSCC related to alcohol consumption and smoking has also begun to decrease. In contrast, the incidence of HPV-associated OPSCC has increased with an increase in HPV infection rates. HPV-associated OPSCC now accounts for approximately 70% of all OPSCC cases in the United States [2, 3]. Similarly, in Japan, the proportion of HPV-associated OPSCC among OPSCC cases is increasing.

Since HPV-associated OPSCC shows markedly favorable oncological behavior compared to classical OPSCC [4], the latest versions of the American Joint committee on Cancer (AJCC) and Union for International Cancer Control (UICC) staging systems classify HPV-associated OPSCC and non-HPV-associated OPSCC separately [5]. Since most HPV-associated OPSCC cases are immunohistochemically positive for p16, HPV-associated OPSCC is classified as p16-positive OPSCC in these classifications. With regard to treatment, although HPV-associated OPSCC is sensitive to chemotherapy and radiotherapy and the prognosis of patients with HPV-associated OPSCC is favorable, it remains unclear whether a reduction in treatment intensity will affect the oncological outcomes. Numerous clinical trials are currently underway to address this issue. From the view point of prevention, it is an urgent issue to promote routine HPV vaccination for both boys and girls at the public expense.

In this review, we provide an overview of HPV-associated OPSCC from the perspectives of epidemiology, carcinogenesis, development, diagnosis, treatment, and prevention.

## Epidemiology

In Europe and the United States, HPV vaccination has resulted in a decrease in cervical cancer, which is the most common HPV-associated cancer. The incidence of cervical cancer decreased by 1.6% annually between 1999 and 2015. Conversely, the incidence of OPSCC increased by 2.7% annually in males and 0.8% annually in females during this time period [6]. Consequently, the incidence of HPV-associated OPSCC is now higher than that of cervical cancer in these regions. The incidence of HPV-associated OPSCC has also gradually increased in Japan. In 1998, Mineta et al. [7] reported HPV-16 positivity in 23% of head and neck cancers and 38% of OPSCCs in Japan. In 2014, Hama et al. [8] reported that 50.3% of OPSCC cases in Japan were HPV-positive. The most recent data from the Head and Neck Cancer Registry in Japan showed that 55% of OPSCC cases were p16-positive [9]. In other Asian countries, the HPV-positive rates in OPSCC also increase, reported to be 70% in South Korea and 67% in China recently [10, 11]. On the other hand, Wu et al. used a Bayesian age-period-cohort model to project the HPV-associated cancer incidence rates of each country up to 2030. While most other Asian countries experience a decreasing or stable trend in HPV-associated cancers incidence rates, Japan and a few countries will have an increasing trend.

Sexual behavior is a well-recognized risk factor for HPV-associated OPSCC, and a strong association between the number of lifetime oral sex partners and the incidence of this disease has been reported [4, 12]. This association may reflect gender differences, as men have been reported to have more sexual partners than women. In addition, the risk of oral HPV infection is associated with a higher number of oral and vaginal sex partners [12]. The HPV-positive rate reported in Mozambique is low, at only 14% [13], which may reflect the fact that oral sexual activity is less common in South Africa. This emphasizes the sexually transmitted nature of oral HPV infection. Drake et al. [14] reported that age at initiation of oral sex, number of partners, presence of children born out of wedlock, and having sexual partners for 10 years or longer were risk factors for OPSCC occurrence, indicating an association between HPV infection and sexual contact.

## Development

Most HPV-associated OPSCCs occur in the palatine and lingual tonsils. This suggests that the specialized histological structure of tonsil tissue is associated with HPV infection. Tonsil tissue consists of a reticulated epithelium lining the crypts. In the reticulated epithelium of the tonsil, epithelial disruption leaves the basal cell layer and basement membrane exposed to viral deposition without mucosal trauma [15]. By contrast, in cervical cancer, HPV infection requires destruction of the epithelium and subsequent deposition of the virus on the exposed basement membrane [16]. As with cervical tissue, tonsil tissue is a histologically favorable environment for HPV invasion and deposition on basal cells.

Integration of HPV DNA into the human genome is also an important step in the carcinogenesis of OPSCC [17]. The oncogenic process of HPV-associated malignancies is primarily mediated by the viral oncoproteins E6 and E7, which bind to and inactivate the tumor suppressors p53 and pRb, respectively. Loss of p53 and Rb results in the loss of cell cycle checkpoints and the inability to undergo apoptosis. HPV is maintained at a low copy number in cells in the basal layer, where immune surveillance occurs. High copy number viral DNA and highly immunogenic capsid proteins do not appear until the virus reaches the superficial differentiation layer of the epithelium, where there are few immune cells. The virus cannot be stimulated without the destruction of host cells or the concomitant induction of an inflammatory response. The absence of a blood-borne stage of HPV infection further limits the exposure of the virus to immune cells. Therefore, HPV-associated OPSCC can escape the antitumor immune response through this mechanism, which must be taken into account when developing treatment strategies for HPV-associated OPSCC [15].

## Clinical features

Neck masses and sore throats are the most common initial symptoms of OPSCC. Patients with HPV-associated OPSCC are more likely to notice a neck mass as the first symptom, whereas patients with classical OPSCC usually complain of sore throat, dysphagia, or odynophagia as the first symptoms [18].HPV-associated OPSCC is often detected at an early stage at the primary site but can also be associated with cervical lymph node metastases [19]. Cervical lymph node metastases of HPV-associated OPSCC are often cystic [20]. The tonsils and base of the tongue are the most common primary sites, accounting for 96% of OPSCC cases [21]. Therefore, when treating primary unknown cervical lymph node metastasis, especially if it shows cystic features, the palatine tonsil and base of the tongue should be checked as possible primary sites.

HPV-associated OPSCC is more sensitive to chemotherapy and radiotherapy than classical OPSCC, and many studies have reported that HPV-associated OPSCC has an extremely favorable prognosis compared to classical OPSCC [22]. In the RTOG0129 study, Ang et al. [23] reported a 3-year overall survival (OS) rate of 82.4% for HPV-associated OPSCC compared to an OS rate of only 57.1% for classical OPSCC and proposed further risk classification by smoking status. However, in Japan, alcohol consumption, not smoking, is the second most prognostic factor after HPV status, possibly due to the low ALDH activity in half of the population [24, 25]. The TAX324 trial, which examined induction chemotherapy for patients with unresectable head and neck cancers, reported 5-year progression-free survival (PFS) rates of 78% and 28% for HPV-associated and classical OPSCC, respectively, with corresponding 5-year OS rates of 82% and 35%, respectively. [26] Notably, the prognosis of patients with HPV-associated OPSCC treated with surgery is also favorable compared to that of patients with classical OPSCC [8].

Given these findings, recent TNM classifications have proposed distinguishing HPV-associated OPSCC from classical OPSCC and classifying HPV-associated OPSCC as an independent disease. Since p16 immunostaining has high sensitivity and specificity for high-risk HPV, such as HPV-16 and HPV-18, and a significant correlation with HPV in situ hybridization [27], p16 immunostaining has been applied as a surrogate marker for HPV status in recent TNM classifications. P16 staining in the nucleus and cytoplasm of 75% of tumor cells or more is considered “p16-positive” in the most recent AJCC guidelines, and staining of 70% of tumor cells or more is considered “p16-positive” in the 6th General Rules for Clinical Studies on Head and Neck in Japan. Primary unknown cervical lymph node metastases positive for p16 are classified as p16-positive OPSCC according to the 8th AJCC TNM classification but as p16-positive primary unknown cancer according to the 8th UICC TNM classification and 6th General Rules for Clinical Studies on Head and Neck, published by the Japan Society for Head and Neck Cancer.

## Treatment strategy

In clinical practice, early OPSCC is treated either by transoral surgery or radiotherapy, and advanced OPSCC is treated by open surgery with reconstruction with or without postoperative CRT or definitive chemoradiotherapy (66–70 Gy radiotherapy with concurrent platinum-based chemotherapy). Transoral robotic surgery (TORS) is a minimally invasive surgical technique for early-stage OPSCC developed by Weinstein et al. in 2007 [28]. Since then, surgical treatment for early-stage HPV-associated OPSCC has increased. The National Cancer Database in the U.S. showed that 68.1% of patients with T1 or T2 HPV-associated OPSCC treated between 2004 and 2013 received surgical treatment [29]. De Almeida et al. [30] reported that 410 patients who underwent TORS had an excellent 2-year local control rate of 91.8%, a disease-specific survival rate of 94.5%, and a crude survival rate of 91%. In that study, 47.3% (160/338) of patients did not receive any postoperative treatment. A prospective study assessing the efficacy of transoral microsurgery in predominantly patients with early-stage (I or II) disease demonstrated 5-year disease-free survival [31], DS, and OS rates of 85%, 93%, and 90%, respectively. [31] Sethia et al. [32] reported that TORS alone led to a higher quality of life (QOL) than adjuvant RT or CRT with respect to eating, social function, speech, and overall postoperative QOL. However, a randomized trial comparing radiotherapy and TORS for OPSCC reported that patients who underwent TORS tended to have slightly worse swallowing function than patients who underwent radiotherapy [33]. This might be because 71% of patients in the TORS group received postoperative radiation therapy. Therefore, when considering the indication of TORS for OPSCC, the possibility of postoperative radiotherapy or chemoradiotherapy, which may result in poor QOL, should be considered in patients with high-risk features, such as multiple cervical lymph node metastases, extranodal spread, and positive or close surgical margins. In other words, if TORS is performed, complete resection with adequate surgical margins is required to maintain QOL. The indications for TORS vary from country to country. Transoral videolaryngoscopic surgery (TOVS) and endoscopic laryngo-pharyngeal surgery (ELPS) have been developed in Japan. The da Vinci Surgical System for TORS was approved by the Pharmaceutical Affairs Agency in August 2018 and covered by national insurance in April 2022 [34]. Sano et al. showed that TORS has an advantage over the other non-robotic modalities in that it has a lower rate of positive surgical margin [35]. Although TORS was gradually used for early OPSCC since then, the number of facilities with TORS remains small in Japan. In South Korea, reports on TORS have been published since around 2008, but the reports were limited from a few facilities, suggesting disparities among facilities [36]. Similarly in China, there are a few reports on TORS. These findings suggest the urgent need to develop robots for transoral surgery suitable for Asian physique and body shape.

## Clinical trials

As patients with HPV-associated OPSCC are more likely to live longer than patients with classic OPSCC and therefore experience late adverse events of definitive chemoradiotherapy [32], there is a great interest in dose reduction of radiation and/or chemotherapy for these patients. Therefore, various attempts have been made to reduce the intensity of CRT, which is the standard non-surgical treatment. However, attempts to reduce treatment intensity should be carefully planned to ensure they do not compromise oncological outcomes. There are considerable difficulties in planning Phase III trials for HPV-associated OPSCC because of the large sample sizes required to demonstrate the non-inferiority of the proposed protocol with reduced intensity, especially in a disease with a favorable prognosis. Of 24 clinical trials on HPV-associated OPSCC [37], 19 (79%) were non-randomized, and 14 employed a phase II design (58%). Only three studies were randomized trials, and only two had a phase III design.

Radiation therapy with cetuximab, dose reduction of postoperative radiotherapy, dose reduction of radiotherapy in CRT, and a combination of radiotherapy with dose reduction and immune checkpoint inhibitors have been assessed to reduce treatment intensity in patients with HPV-associated OPSCC. The RTOG1016 trial was designed to test the non-inferiority of bioradiotherapy (BRT) using cetuximab to CDDP-based CRT [38], but BRT failed to prove its non-inferiority to CDDP-based CRT and did not reduce the incidence of adverse events. Similarly, the De-ESCALaTE trial [39] failed to demonstrate a decrease in the number of adverse events. In addition, the survival rate of the BRT group was significantly lower than that of the CRT group. These results indicate that CDDP-based CRT should be the standard of care for patients with locally advanced HPV-associated OPSCC.

Treatment de-escalation for patients at a high risk of recurrence should be avoided, even those with HPV-associated OPSCC. In the NRG Oncology HN002 study [40], 60 Gy of radiotherapy with low-dose CDDP in low-risk patients (T1–T2/N1–N2b or T3/N0–N2b disease) had a similar PFS and a lower incidence of Grade 3–4 adverse events compared to historical controls treated with standard CRT with CDDP. This group is currently conducting another study (HN005) to evaluate the efficacy of low-dose radiation therapy and immune checkpoint inhibitors. The ECOG 1308 trial was a phase II trial of chemoselection with induction chemotherapy using paclitaxel, CDDP, and cetuximab, with a radiation dose reduction to 54 Gy in patients who achieved CR. The low-dose group had an acceptable 2-year OS rate of 94%, with significantly fewer cases of dysphagia and nutritional problems. Survival was particularly favorable in the low-risk group. Therefore, appropriate use of induction chemotherapy, especially among patients at low risk of recurrence, may allow for radiation dose reduction and a favorable post-treatment quality of life. In the ECOG3311 trial [41], the authors attempted to reduce the dose of RT after transoral surgery. For patients with low-risk disease, the 2-year PFS of patients who did not receive postoperative treatment was favorable. Further, Yang et al. [42] conducted a meta-analysis of RT reduction in HPV-associated OPSCC. The 3-year OS rate was higher in the reduced-dose group than in the standard-dose group (91.5% vs. 87.46%). Therefore, for patients with uninvolved surgical margins, < 5 involved nodes, and minimal ( < 1 mm) ENE, reduced-dose postoperative RT without chemotherapy is sufficient. [41]

## Vaccination

HPV vaccination has largely been promoted to prevent cervical cancer. Accordingly, in Western countries with high rates of vaccination, the incidence of cervical cancer has decreased [43–45]. By contrast, in Japan, the vaccination rate is extremely low compared to other countries owing to issues raised by the adverse reactions of HPV vaccination [46, 47], which has led to a significant increase in HPV infection rates in young Japanese individuals.

Since the average age at which patients are affected by OPSCC is significantly higher than that of patients with cervical cancer, it would take several decades to verify the efficacy of HPV vaccination in preventing HPV-associated OPSCC. Therefore, there is currently no clear evidence that HPV vaccination prevents HPV-associated OPSCC. However, oral HPV infection might be used as a surrogate. Studies on oral HPV infection rates among Japanese healthcare workers have shown that HPV infection rates are higher among men in their 30s and older [48]. In addition, HPV vaccination has been reported to reduce the rate of oral HPV infection [49]. The most common genotype, HPV-16, and the majority of other high-risk genotypes detected in HPV-associated OPSCC are covered by the 4-valent and 9-valent HPV vaccines [50, 51]. These findings indicate that HPV vaccination before sexual contact in both boys and girls may prevent OPSCC.

Several countries have implemented national HPV vaccination programs for girls and boys. Australia was one of the first countries to implement a program for boys and girls and demonstrated high levels of vaccine administration beginning in 2013, with 75.9% and 80.2% vaccination rates for boys and girls, respectively, in 2017 [52]. Therefore, reports on the efficacy of HPV vaccination in preventing HPV-associated OPSCC in these countries are required.

Several studies have estimated the future incidence of HPV-associated OPSCC based on vaccine coverage. Damgacioglu et al. [53] estimated that if a 50% vaccination rate could be achieved, the incidence of OPSCC would be halved in approximately 40 years, and if a rate of 80% could be achieved, the incidence would be approximately 1/5 the current rate. Brisson et al. [54] estimated that HPV eradication would be possible if the vaccination rate for both men and women was maintained at 80%. Routine HPV vaccination of both boys and girls is desirable. Landy et al. reported a microsimulation-based, individual-level, state-transition model of oral HPV-16 infection and HPV-16-positive oropharyngeal cancer among heterosexual US men aged 15–84 years and found that, in the absence of vaccination, most (70%) causal oral HPV-16 infections were acquired by age 26, and 29% were acquired between ages 27–45. Therefore, the vaccine should be administered before the age of 26 years and is unlikely to be effective if administered after the age of 27 years.

## Conclusion

HPV-associated oropharyngeal carcinoma is one of the few carcinomas increasing in incidence. In Japan, the incidence of HPV-associated OPSCC is expected to continue to increase over the long term, partly because of the prolonged suspension of the HPV vaccination program. It is important to prevent HPV-associated OPSCC by promptly establishing a public vaccination program including both boys and girls and by educating the public about HPV infection and HPV-associated cancers. Because the prognosis of patients with HPV-associated OPSCC is favorable, large-scale phase III clinical trials are needed to develop treatments aimed at reducing treatment intensity. Promising clinical trial results have been reported on reducing radiation doses in low-risk patients and reducing the intensity of postoperative radiotherapy after transoral resection. Careful decisions must be made to ensure that a reduction in treatment does not decrease oncological outcomes.
